# Determinants of health-seeking behaviors among middle-aged women in Vietnam's rural-urban transition setting

**DOI:** 10.3389/fpubh.2022.967913

**Published:** 2023-01-10

**Authors:** Thao Thi Phuong Nguyen, Cuong Tat Nguyen, Hieu Trung Do, Ha Thai Tran, Thuc Minh Thi Vu, Son Nghiem, Giang Thu Vu, Carl A. Latkin, Cyrus S. H. Ho, Roger C. M. Ho

**Affiliations:** ^1^Institute for Global Health Innovations, Duy Tan University, Da Nang, Vietnam; ^2^Faculty of Medicine, Duy Tan University, Da Nang, Vietnam; ^3^Faculty of Medicine, Hung Yen Medical College, Hung Yen, Vietnam; ^4^Department of General Planning, National Hospital of Traditional Medicine, Hanoi, Vietnam; ^5^Institute of Health Economics and Technology, Hanoi, Vietnam; ^6^Center for Applied Health Economics, Griffith University, Brisbane, QLD, Australia; ^7^Center of Excellence in Evidence-Based Medicine, Nguyen Tat Thanh University, Ho Chi Minh City, Vietnam; ^8^Bloomberg School of Public Health, Johns Hopkins University, Baltimore, MD, United States; ^9^Department of Psychological Medicine, National University Hospital, Singapore, Singapore; ^10^Department of Psychological Medicine, National University Health System, Singapore, Singapore; ^11^Department of Psychological Medicine, Yong Loo Lin School of Medicine, National University of Singapore, Singapore, Singapore; ^12^Institute for Health Innovation and Technology (iHealthtech), National University of Singapore, Singapore, Singapore

**Keywords:** middle-aged, menopause, chronic disease, health-seeking, health service

## Abstract

**Introduction:**

The purpose of this study is to identify the health status and healthcare utilization factors associated with middle-aged women in the rapid urbanization context of Vietnam.

**Methods:**

A cross-sectional study with a systematic random sampling technique was conducted in Hung Yen city. A systematic random sampling technique was used to select a sample size of 362 middle-aged women. The collected data included socioeconomic characteristics, health issues, health service utilization, and social support for women in both urban and rural areas. Multivariate regression models were used to determine factors associated with health service utilization and the number of inpatient/outpatient visits.

**Results:**

Among 362 participants, the main chronic diseases were diabetes (12.8%), cardiovascular diseases (11.3%), and migraines (9.5%). The proportion of using inpatient and outpatient services among middle-aged women was relatively high (35.8 and 61%, respectively). Women having more support from family and friends were less likely to seek healthcare. Living in rural areas significantly increased the number of inpatient treatments. Regarding health service utilization, the percentage of people using outpatient treatment services accounted for 61.0%, while using inpatient treatment services was reported as 35.8%. The average number of outpatient and inpatient visits per participant were 1.3 and 0.9, respectively.

**Conclusion:**

This study revealed a relatively high rate of using healthcare services among women at midlife in a rapidly industrializing city in Vietnam. Screening programs should be implemented for early detection and treatment of chronic diseases in middle-aged women, especially for diabetes. Communication strategies should be adapted to raise awareness of rural women about regular health checkups, and counseling services of healthcare providers should be strongly reinforced. Lifestyle interventions and health promotion programs involving social support should be implemented to improve wellbeing and healthcare-seeking behaviors among middle-aged women.

## 1. Introduction

After the independence declaration in 1945, Vietnam's healthcare system was first established in the Northern; subsequently, the system was expanded to the Southern after the nation's unification in 1975. Prior to 1989, the healthcare system was mainly organized and wholly financed by Vietnam's government; thus, people were provided free healthcare services in this period (1). Along with the reformed economy, there was a transformation in the healthcare system from a fully public service system to a combined public-private provider system since 1989 (2). Furthermore, since its inception in 1992, health insurance has offered financial security for people who use healthcare services as well as funding for the healthcare system's development (2). Regarding the disease burden in Vietnam, non-communicable diseases accounted for 72% of the overall, which included cardiovascular diseases, particularly stroke, and unintentional injuries (3). In women, depression and stroke were the leading causes of disease burden according to Disability-adjusted life years (DALYs), which is higher than in men. Moreover, among middle-aged women, non-communicable diseases such as depression and stroke were the key causes of disease burden (4). Furthermore, exposure to particular hazards, such as tobacco, alcohol, unsafe sex, or poor sanitation, can significantly increase individuals' risks of developing the disease in women, especially middle-aged women (5).

Middle age is a unique transitional life phase and presents with its distinctive sets of problems. Middle-aged women often encounter a wide range of both physical and psychological health problems when approaching menopause (6, 7), most commonly vasomotor symptoms, mood changes, sleep disorders, and sexual dysfunction (8). In addition to the psycho-physiological symptoms, various chronic conditions also occur frequently among women during middle age (9). Severe vasomotor symptoms and sleep disorders might lead to the onset of cardiovascular diseases, while depression in combination with severe vasomotor symptoms may have negative effects on cognitive functions (10). Menopausal symptoms can become distressing and affect the personal, interpersonal, and community lives of women considerably (10, 11). Negative effects of menopausal symptoms on women's quality of life have been well-evident in previous studies (12–14). Currently, Vietnam has few studies referring to health-seeking behavior and how it should be measured for the targeted group of middle-age women. Several existing literatures have documented health care-seeking behaviors among middle-aged women in developing countries with rapid rural-urban transition similar to Vietnam. For instance, in India, the percentage of women seeking treatment for their problems was higher in the urban areas, i.e., 71.0% as compared to 29.5% in rural areas (15). In Nigeria, most menopausal women tended to consult pharmacy staff (51.4%) or health workers (44.7%). Similar findings have been reported in Bangladesh, where the majority of the participants seek healthcare mostly from local doctors, followed by general practitioners and traditional healers (12–14).

Some factors associated with health service utilization among middle-aged women have been investigated in previous studies. A recent study in China concluded that higher age, health insurance status, employment status, various symptoms, sleep disorders, urinary incontinence, and non-specialized department visits were significantly correlated with longer delays in approaching perimenopausal healthcare (16), while another study conducted in North-East Malaysia among menopausal women with urinary incontinence (UI) identified age of menopause and severity of UI as factors associated with seeking treatment (17). Although both studies emphasized the influence of socioeconomic characteristics and health conditions on health service utilization, they did not consider key determinants such as social support and their substantial effects on healthcare-seeking behaviors among mature women.

Although rapid industrialization has promoted socioeconomic development and increased access to health services worldwide, it has also resulted in various environmental and health problems. Therefore, ensuring healthcare services for vulnerable groups, such as middle-aged women, is critical to maintaining quality of life during globalization. Understanding health conditions and health service utilization of vulnerable populations is vital to facilitate appropriate interventions. Despite its important implications, little is known about healthcare-seeking behavior among middle-aged women in Vietnam. Therefore, the purpose of this study is to identify the health status, healthcare access, as well as their associated factors, among middle-aged Vietnamese women.

## 2. Methods

### 2.1. Study setting, samples, and sampling

#### 2.1.1. Study setting

A cross-sectional study was carried out from June to December 2018 in Hung Yen city. This place is a large metropolis in Northern Vietnam, with diverse socioeconomic characteristics, including urban and rural areas.

#### 2.1.2. Samples and sampling

Three hundred and sixty-two middle-aged women were recruited for the current study. The eligibility criteria for selecting participants were: (1) aged 40–60 years old; (2) residing with a local household registration in Hung Yen during the survey. Participants were excluded from the study selection if they suffered from severe illnesses during the interview period.

**Figure 1:**
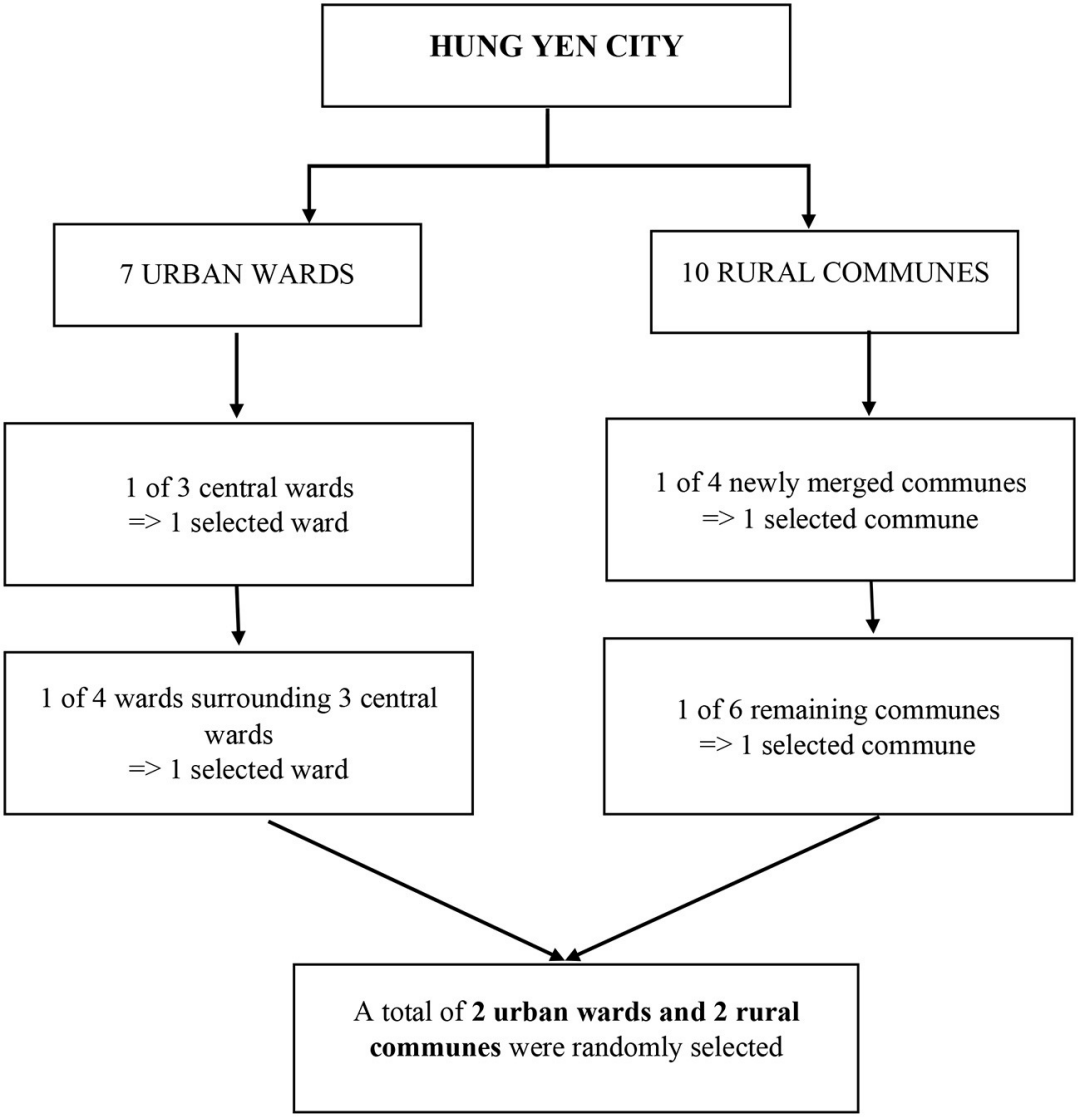
Systematic random sampling technique in the study.

A systematic random sampling technique was used to select a sample size of 362 middle-aged women. Hung Yen City had seven urban wards and 10 rural communes. We randomly selected one of three central wards and one of four wards surrounding three central wards. One of four newly merged communes and one of six remaining communes were randomly chosen. In total, there were four wards/communes selected in the study. A total of two urban wards and two rural communes were randomly selected in the current study (Figure 1). Information of 100 participants was targeted for collection in each ward/commune. A sample frame of all middle-aged women in each ward/commune was provided by the local authorities. We used systematic sampling to select participants (*k* = 7). If a middle-aged woman with the selected ordinal number was absent at the health examination, a middle-aged woman with the next ordinal number would be selected instead. This sample size was calculated using a power of 0.8, a significance level of 0.05, and a conservative Cohen's effect size of 0.025 (18).

All participants were invited into a private counseling room in the health commune stations to secure their confidentiality. Participants were informed of the objectives, advantages, and drawbacks of the study. Hundred percent of respondents agree to participate in this study by signing a written consent.

### 2.2. Measures and instruments

A 15–20 min interview was carried out using a structured questionnaire, which consists of four main components, including (1) Demographic characteristics; (2) Health status; (3) Social support; (4) Health service utilization. All interviewers had undergone a 2-day intensive training session before the survey. To ensure the text and logical issues before collecting data, the questionnaire was piloted on 10 people of different ages and occupations.

#### 2.2.1. Covariates

*Demographic characteristics*: Respondents' background information included (1) age group (40–44, 45–49, 50–54, and 55–60); (2) marital status (single/divorced/widow, and married); (3) occupation (farmers, blue-collar workers, white-collar workers, business owners, house-makers, retirees, others); (4) income quintiles (very low, low, medium, high, and very high), and (5) monthly income (USD).

*Health status*: Participants were asked to self-report chronic conditions that they had been diagnosed with in the last 3 months, including (1) health issues (migraine, diabetes, and cardiovascular disease) and (2) medicine use (none, one medicine, and more than one medicine).

*Health information sources*: This was received from friends/relatives, posters/banner, internet, text messages, radio/television, community loudspeakers, newspapers/books, medical staff, and social networking.

*Social support*: The Multidimensional Scale of Perceived Social Support (MSPSS) was utilized to assess the support from spouses, family, and friends. The tool consisted of 12 questions divided into three groups of support from (1) Significant other (four questions), (2) Family (four questions), and (3) Friends (four questions). Each question was rated from 1 “strongly agree” to 7 “strongly disagree” (19). The total score of social support ranged from 1 to 7. The higher overall score indicated higher social support. Moreover, MSPSS has been widely utilized and validated in the Vietnamese population (20–22).

#### 2.2.2. Outcome variables

##### Health service utilization

Participants were asked to self-report on (1) using inpatient and/or outpatient services (yes or no) during the last 12 months; and (2) the number of inpatient and/or outpatient treatments.

### 2.3. Data analysis

The STATA 12.0 software (Stata Corp. LP, College Station, TX, USA) was used to analyze the data. Continuous variables were displayed as mean and standard deviation (SD), whereas categorical variables were shown as frequency with percentage. Demographic characteristics, health status, social support, and health service utilization. The differences in socioeconomic characteristics, healthcare service utilization, and health information-seeking behavior between urban and rural areas were described using the Chi-square test and ANOVA test. Multivariate logistic regression models were used to determine factors associated with using inpatient/outpatient treatments (yes or no). Meanwhile, multivariate regression was utilized to determine factors related to the number of inpatient/outpatient treatments (quantitative variable). The potential covariates for the full model were the demographic characteristics, health status (health issues and medicine use), health information sources, and social support. A *p*-value < 0.05 was considered statistically significant.

### 2.4. Ethical consideration

This study protocol was reviewed and approved by the Scientific Committee of the Youth Research Institute, Ho Chi Minh Communist Youth Union (Code 177/QD/TWDTN-VNCTN).

## 3. Results

Table 1 presents the demographic characteristics of participants. Among 362 respondents in our study, 50.3% lived in urban areas. Most of the participants were living with spouses (88.7%). Among occupations, farmers accounted for the highest proportion (42.0%), followed by business (14.5%), and blue-collar workers (10.8%). There is a difference in income quintiles between participants in urban and rural areas. The majority of participants who live in urban areas reported having high incomes (27.9%), while women living in rural areas revealed that most of them have low incomes (34.7%). The mean age and monthly incomes were 49.6 ± 5.9 years and $272.5 ± $212.9, respectively. The significant differences between urban and rural areas were found in the variable groups, including age group, occupation, and income quintiles (*p* < 0.01).

**Table 1:** Characteristics of participants.

**Characteristics**	**Urban area**		**Rural area**		**Total**		***p*** **-value**
	***N***	**%**	***n***	**%**	***n***	**%**	
**Total**	201	50.3	199	49.8	400	100.0	
**Age group**							
40–44	44	21.9	62	31.2	106	26.5	< 0.01
45–49	34	16.9	60	30.2	94	23.5	
50–54	68	33.8	27	13.6	95	23.8	
55–60	55	27.4	50	25.1	105	26.3	
**Marital status**							
Single/divorced/widow	28	13.9	17	8.6	45	11.3	0.092
Married	173	86.1	181	91.4	354	88.7	
**Occupation**							
Farmers	69	34.3	99	49.8	168	42.0	< 0.01
Blue-collar workers	33	16.4	10	5.0	43	10.8	
White-collar workers	17	8.5	24	12.1	41	10.3	
Business owners	22	11.0	36	18.1	58	14.5	
House-makers	25	12.4	13	6.5	38	9.5	
Retirees	16	8.0	16	8.0	32	8.0	
Others	19	9.5	1	0.5	20	5.0	
**Income quintiles**							
Very low	40	19.9	46	23.1	86	21.5	< 0.01
Low	52	25.9	69	34.7	121	30.3	
Medium	24	11.9	10	5.0	34	8.5	
High	56	27.9	23	11.6	79	19.8	
Very high	29	14.4	51	25.6	80	20.0	
	**Mean**	**SD**	**Mean**	**SD**	**Mean**	**SD**	***p*** **-value**
**Age (years)**	50.4	5.5	48.7	6.2	49.6	5.9	0.01
**Monthly income (USD)**	261.1	199.1	284.0	225.9	272.5	212.9	0.83

Table 2 showed the health status, health information sources, and health service utilization, of participants. The percentages of the three most common diseases include diabetes, migraine, and cardiovascular disease, which were reported, respectively at 12.8, 11.3, and 9.5%. The percentage of cardiovascular disease was highest among urban women (16.9%), while this figure was highest for diabetes among rural women (14.6%). The statistically significant difference between rural and urban areas was found in the groups of migraine and cardiovascular disease (*p* ≤ 0.01). Regarding health service utilization, the percentage of people using outpatient treatment services accounted for 61.0%, while using inpatient treatment services was reported as 35.8%. The average number of outpatient and inpatient visits per participant were 1.3 and 0.9, respectively. Three-fifth of the sample reported using only one medicine (58.0%). The most common channels to receive health information were radio/television (98.8%) and community loudspeakers (48.8%).

**Table 2:** Health status, health information sources, and health service utilization of participants.

**Characteristics**	**Urban area**		**Rural area**		**Total**		***p*** **-value**
	***N***	**%**	***n***	**%**	***n***	**%**	
**Health issues**							
Migraine	11	5.5	27	13.6	38	9.5	0.01
Diabetes	22	11.0	29	14.6	51	12.8	0.28
Cardiovascular disease	34	16.9	11	5.5	45	11.3	< 0.01
**Medicine use**							
None	49	24.4	43	21.6	92	23.0	0.52
One medicine	118	58.7	114	57.3	232	58.0	
More than one medicine	34	16.9	42	21.1	76	19.0	
**Health information sources**							
Friends/relatives	16	8.0	14	7.0	30	7.5	0.73
Posters/banner	1	0.5	3	1.5	4	1.0	0.31
Internet	27	13.4	29	14.6	56	14.0	0.74
Text message	4	2.0	10	5.0	14	3.5	0.10
Radio/television	199	99.0	196	98.5	395	98.8	0.65
Community loudspeaker	98	48.8	97	48.7	195	48.8	1.00
Newspapers/books	37	18.4	24	12.1	61	15.3	0.08
Medical staff	21	10.5	20	10.1	41	10.3	0.90
Social network	5	2.5	5	2.5	10	2.5	0.99
**Using inpatient treatments**	64	31.8	79	39.7	143	35.8	0.10
**Using outpatient treatments**	130	64.7	114	57.3	244	61.0	0.13
	**Mean**	**SD**	**Mean**	**SD**	**Mean**	**SD**	***p*** **-value**
**Number of inpatient treatments**	0.5	0.9	0.6	0.9	0.6	0.9	0.06
**Number of outpatient treatments**	1.3	1.4	1.1	1.2	1.2	1.3	0.13

Table 3 reveals the multivariate regression model using outcomes variables was using inpatient/outpatient treatment services (yes/no) and the number of inpatient/outpatient visits. People living in urban areas were more likely to use inpatient treatment services as compared to those in rural areas (OR = 2.15, 95% CI: 1.26; 3.67, *P* < 0.001). Business owners (OR = 6.04, 95% CI: 2.89; 12.64, *P* < 0.001), house-makers (OR = 2.51, 95% CI: 1.03; 6.08, *P* < 0.05) were more likely to use inpatient treatment services as compared to farmers. Meanwhile, blue-collar workers (OR = 7.32, 95% CI: 2.50; 21.42, *P* < 0.001), business owners (OR = 5.82, 95% CI: 2.69; 12.59, *P* < 0.001), and other occupation groups (OR = 7.61, 95% CI: 2.16; 26.84, *P* < 0.001) tend to use outpatient treatment services more than the farmer group. Women with high incomes were more likely to attend outpatient with 2.98 times increased odds compared to the lowest income group (95% CI = 1.50; 5.92, *P* < 0.001).

**Table 3:** Associated factors with health service utilization.

**Characteristics**	**Using inpatient treatments**		**Number of inpatient treatments**		**Using outpatient treatments**		**Number of outpatient treatments**	
	**aOR**	**95% CI**	**Coef**.	**95% CI**	**aOR**	**95% CI**	**Coef**.	**95% CI**
**Socioeconomic characteristics**								
Marital status (living with spouse vs. single/divorced/widow)	0.35^**^	0.14; 0.91	−0.51^**^	−0.94; −0.08	2.48^*^	0.91; 6.74	0.28	−0.07; 0.64
Living area (rural vs. urban)	2.15^***^	1.26; 3.67	0.40^**^	0.09; 0.71				
**Occupation (vs. farmers)**								
Blue-collar workers					7.32^***^	2.50; 21.42	0.63^***^	0.34; 0.92
Business owners	6.04^***^	2.89; 12.64	1.07^***^	0.68; 1.47	5.82^***^	2.69; 12.59	0.63^***^	0.31; 0.95
House-makers	2.51^**^	1.03; 6.08	0.70^***^	0.26; 1.14			0.46^**^	0.10; 0.82
Retirees	2.53^*^	0.95; 6.74	0.95^***^	0.48; 1.42			0.40^**^	0.03; 0.77
Others	2.3	0.71; 7.42			7.61^***^	2.16; 26.84	0.82^***^	0.37; 1.27
**Income quintiles (vs. lowest)**								
Lower							−0.63^***^	−0.96; −0.30
Medium							−0.23^**^	−0.46; −0.00
High					2.98^***^	1.50; 5.92		
Highest					2.01	0.87; 4.65		
**Age**	1.06^**^	1.01; 1.12	0.04^***^	0.01; 0.07	1.04^*^	1.00; 1.09	0.03^**^	0.01; 0.04
**Health status**								
**Health issues (yes vs. no)**								
Migraine	3.17^**^	1.27; 7.92	0.73^***^	0.30; 1.16	2.83^**^	1.18; 6.80	0.48^***^	0.13; 0.83
Diabetes	2.44^**^	1.10; 5.40	1.14^***^	0.79; 1.50	77.97^***^	10.15; 598.97	1.24^***^	1.02; 1.46
Cardiovascular disease	5.44^***^	2.36; 12.57	1.10^***^	0.66; 1.53	15.14^***^	5.33; 43.02	1.23^***^	0.97; 1.49
**Medicine use (vs. none)**								
One medicine	0.49^**^	0.26; 0.94						
More than one medicine	0.57	0.25; 1.30						
**Social support**								
**Perceived Social Support (MSPSS)**								
Spouse	3.00^***^	1.89; 4.78	0.64^***^	0.38; 0.90	0.95	0.64; 1.39		
Family	0.58^**^	0.35; 0.94	−0.46^***^	−0.74; −0.17	0.57^**^	0.35; 0.92	−0.08	−0.20; 0.05
Friends	0.59^***^	0.43; 0.81	−0.21^**^	−0.42; −0.01			−0.14^**^	−0.26; −0.03

*** *p* < 0.01,

** *p* < 0.05,

* *p* < 0.1.

aOR, adjusted OR.

Participants who had health issues such as migraines, diabetes, and cardiovascular disease were more likely to use health services than those who did not. Moreover, women who had more support from their spouses were more likely to use the inpatient treatment services (OR = 3.00, 95% CI: 1.89; 4.78, *P* < 0.001). Meanwhile, participants who received support from family and friends were less likely to seek healthcare.

## 4. Discussion

Our study provides insights into health conditions and health service utilization among middle-aged women in Hung Yen, a major city undergoing rapid urbanization and population growth in Vietnam. The findings of this study highlighted common chronic diseases as well as the high rate of inpatient/outpatient services in middle-aged women. We also discovered primary sources of health information and associated factors, as well as important implications for improving health-related quality of life among women during menopause.

Vietnam is a developing nation with a high burden of non-communicable diseases, which accounted for 72% of total deaths (3). Participants reported that the three main chronic conditions that they were diagnosed with in the last 3 months were diabetes, cardiovascular diseases (CVD), and migraines. These health problems are among the key issues with the most devastating burdens on middle-aged women in terms of disability, morbidity, mortality, and quality of life (23). Previous literature in other countries also demonstrated a higher risk of diabetes, CVD, and migraine among women at menopausal age (24–26). The prevalence of diabetes among middle-aged women in this study (12.8%) was higher than that in the general population in Vietnam (4.9%) (27), suggesting the need for more efforts to prevent diabetes among this population. Notably, in this research, women in rural areas had a higher rate of migraines but a lower proportion of having CVD than those in urban areas (13.6–5.5% and 5.5–16.9%, respectively). These results called for further studies to examine the discrepancy in the prevalence of the two above-mentioned diseases in different settings.

Our research indicated high rates of using healthcare services among mid-aged women, with 61% registered as outpatients, and 35.8% as inpatients. These figures were higher than those of the general Vietnamese population (37.1% outpatient and 8.1% inpatient visits) according to an analysis of Vietnam living standard survey data (28), and other specific populations such as farmers in mountainous areas (50.3% used outpatient and 28.9% used inpatient cares) (29). This discrepancy might be explained by different levels of health literacy among subjects and the accessibility to health facilities in different settings. Since respondents in our study live in a developed city with a convenient transportation system, they were able to seek healthcare services more easily than the farmers living in rural areas with limitations on transportation due to long distances and geological hazards. A study in the United States reported similar findings, which indicated that 60% of respondents used health services to treat their menopausal symptoms, and more than 50% of these women sought health care within the past 12 months (30). A lower rate of healthcare utilization among menopausal women was also reported in a study of 386 Australian participants, in which 30% of women visited a doctor annually during the menopausal transition to seek health advice (31). Another remarkable finding from our study was the disparity in the pattern of health service usage among urban and rural participants. A previous study in India has demonstrated that the percentage of mature women seeking treatment was higher in the urban areas, i.e., 71.0% as compared to 29.5% in rural areas (15). Our research has more specific results, which point out that women living in lower resource settings had a higher rate of using inpatient treatments but a lower rate of using outpatient treatments than those in urban areas. This difference could be attributed to the variation of healthcare-seeking behaviors among those two groups. Indeed, the majority of women in rural areas go to health facilities only in the case of severe illness or at the late stage of disease, while in contrast, urban participants may have more frequent health checkups to reduce the risk of hospitalization (32).

Most of our respondents accessed health information through mass media such as television/radio (98.8%) and community loudspeakers (48.8%). Only 10.3% of participants received health advice from medical staff, lower than those seeking information from the Internet (14%). This finding highlights the need to strengthen the counseling services for mature women. A study in the United States (US) showed a different pattern: most of their study subjects preferred receiving health information from health workers (62.7%), and the Internet and mass media only accounted for 13.5 and 12.2% (33). This disparity might be due to the difference in health systems in the two countries. As family physicians offer majority services to underserved rural and urban communities in America (34), US citizens can easily seek health advice from them. On the contrary, the family medicine workforce in Vietnam is still in its infancy, making Vietnamese people more likely to encounter barriers to reliable information from healthcare providers.

Factors associated with health service utilization among menopausal women have also been investigated in several studies. Recent research in Shanghai, China concluded that higher age, having no health insurance, employment status, various symptoms, sleep disorders, urinary incontinence, and non-specialized department visits were significantly correlated with a longer delay in approaching perimenopausal healthcare (16). In another study conducted in North-East Malaysia, among menopausal women with urinary incontinence (UI), factors associated with seeking treatment were the age of menopause and severity of UI (17). In comparison to the above research, our study pointed out a new aspect that significantly influenced the use of healthcare among middle-aged women–social support. According to our multivariate regression model, people who had more support from family and friends were less likely to seek healthcare services. This evidence suggests a new research trajectory that focuses on social support as a health care provider and the influence of social support on the utilization of healthcare among mature women.

Several implications can be drawn from this study. As chronic conditions are prevalent among middle-aged women, screening programs should be implemented for early detection and treatment of these diseases, especially for diabetes. In addition, further research should be conducted to examine the differences between the prevalence of migraine and CVD in urban and rural areas and propose appropriate interventions for particular settings. The high rate of health services utilization among middle-aged women may result in a significant burden on the health system. Therefore, public health practitioners should focus on lifestyle interventions for females with menopausal symptoms to reduce the burden on the system. To reduce the risk of hospitalization for women in rural areas, communication strategies should be adapted to raise their awareness of the importance of regular health checkups. Moreover, traditional channels such as TV/radio and community loudspeakers should continue to provide reliable health information for middle-aged women, alongside with the reinforcement of counseling services from medical staff. Finally, future health promotion programs should involve social support from family and friends to improve wellbeing and healthcare-seeking behaviors among women at mid-life.

The interpretation of our study's findings should be acknowledged with the following limitations. Firstly, as the cross-sectional study design was adopted, we were unable to conclude the causal relationship between health service utilization and its determinants. Secondly, data was collected in one province in Northern Vietnam, thus our results could not be representative of other regions. Nevertheless, this research can still offer valuable reference for policy development toward middle-aged women with similar social settings. Thirdly, because the information was self-reported by middle-aged women, recall bias could occur. Fourth, some of the participants may have specific health issues but have not been diagnosed yet, which might lead to an underestimation of their morbidities, such as depression, a female-predominant condition with a global lifetime prevalence of 10.8% (35). Depression is also associated with diabetes (36) and cardiovascular diseases (37). Due to stigma (38), middle-aged women were less likely to report depression. Despite the limitations, the research team optimized the significance of this study by applying systematic random sampling and training healthcare workers to collect high-quality data. The heterogeneity of this study population was also improved as we surveyed both urban and rural areas.

## 5. Conclusion

This study emphasized a high proportion of healthcare services usage among middle-aged women in a rapidly urbanizing city in Northern Vietnam. Screening programs should be implemented for early detection and treatment of chronic diseases in mature women, especially for diabetes. Communication strategies should be adapted to raise awareness of rural women on regular health checkups, and the counseling services of healthcare providers should be strongly reinforced. Public health professionals should consider implementing lifestyle interventions aiming at improving quality of life among females with menopausal symptoms and mitigating the burden on the health system. Future health promotion programs should involve social support to improve wellbeing and healthcare-seeking behaviors among women at mid-life.

## Data availability statement

The raw data supporting the conclusions of this article will be made available by the authors, without undue reservation.

## Ethics statement

The studies involving human participants were reviewed and approved by Scientific Committee of Youth Research Institute, Ho Chi Minh Communist Youth Union. The patients/participants provided their written informed consent to participate in this study.

## Author contributions

TN, HD, CL, and CH: conceptualization. CN, HD, TV, and HT: data curation. HD, TV, SN, and CL: formal analysis. CN, HT, GV, and RH: investigation. HD, TV, and SN: methodology. GV, CH, and RH: supervision. TN, CN, and SN: writing—original draft. TN, HD, HT, GV, CL, CH, and RH: writing—review and editing. All authors contributed to the article and approved the submitted version.
